# Propensity matching cannot substitute for randomization in albumin studies

**DOI:** 10.1186/s13054-015-0786-z

**Published:** 2015-04-28

**Authors:** Christian J Wiedermann, Wolfgang Wiedermann

**Affiliations:** Department of Internal Medicine, Central Hospital of Bolzano/Bozen, Teaching Hospital of the Medical University of Innsbruck, Lorenz-Böhler Street 5, 39100 Bolzano/Bozen (BZ), Italy; Department of Psychology, Unit of Quantitative Methods, University of Vienna, Liebiggasse 5, 1010 Vienna, Austria

Large randomized trials such as the Albumin Italian Outcome Sepsis (ALBIOS) trial involving 1,818 patients with severe sepsis have revealed no evidence of acute kidney injury (AKI) attributable to albumin infusion [1]. Such results are difficult to reconcile with an association between albumin and AKI in the retrospective study by Frenette and colleagues of 984 cardiac surgery patients receiving 6% hydroxyethyl starch 130/0.4, 10% pentastarch, 5% albumin and/or 25% albumin in unspecified combinations [2]. Those investigators criticize the ALBIOS trial for a lack of data on timing of AKI in relation to albumin infusion, but that is also among the shortcomings of their own study. Baseline data are not stratified by colloid group, so imbalances cannot be assessed. The dose–response analysis is univariate only. The propensity matching did not include known independent risk factors for AKI such as preoperative hypoalbuminemia [3] and cardiac catheterization [4]. Furthermore, the matching failed to achieve satisfactory balance, since there remained a significant difference in concomitant pentastarch dose.

Frenette and colleagues misinterpret a study on the robustness of propensity scores [5] as suggesting that their study may have underestimated AKI risk associated with albumin infusion. The study showed that mortality was decreased by albumin in randomized trials of critically ill patients with an odds ratio of 0.82 and a 95% confidence interval of 0.67 to 1.00, but mortality was increased with propensity score matching of observational study data (odds ratio, 2.02; confidence interval, 1.43 to 2.84). The difference was significant (ratio of odds ratio, 0.43; confidence interval, 0.29 to 0.63). Thus, propensity scores evidently cannot overcome the tendency to use albumin as salvage treatment in sicker patients.

## Abbreviations

AKI: Acute kidney injury
ALBIOS: Albumin Italian Outcome Sepsis
