# Supplementary material for: Propensity matching cannot substitute for randomization in albumin studies
Source: Crit Care. 2015 Apr 28;19(1):206. doi: 10.1186/s13054-015-0786-z (PMC4411655; doi:10.1186/s13054-015-0786-z)
Supplement: Related texts

# Reply: Authors’ response

**Authors:** Josée Bouchard, Anne Julie Frenette, Stéphan Troyanov, David R Williamson

We agree with Wiedermann and Wiedermann that propensity scores will never replace randomized controlled trials. However, the study design of the ALBIOS trial differed from our study on several points [1]. In the ALBIOS trial, albumin was administered to patients with hypoalbuminemia and severe sepsis or shock according to albumin levels, and not according to the clinical context. In addition, the mean daily dose of albumin administered was substantially lower in this randomized trial (0.4 mg/kg/day over the first 7 days vs 0.9 mg/kg/day in our study) and the period of administration was much longer than our study (28 days vs 36 hours in our study) [1,2]. The timing of AKI in relation to albumin administration was not detailed in the ALBIOS trial, while we summarized these results in a figure and in the text.

Regarding our study, the unbalanced results from the matching of our propensity score would have biased the results toward a decreased AKI rate in the albumin group. In addition, the percentage of patients undergoing cardiac catheterization was similar between the no albumin and albumin groups in the propensity score (27.0% vs 25.5%, *P* = 0.79). As mentioned, our study does not provide a definitive answer on the use of albumin in patients undergoing cardiac surgery. Future studies should be performed on the use of albumin in surgical populations.
